# SELF-EMPLOYMENT AMONG OLDER HISPANICS: INTRAGROUP DIVERSITY, WORKPLACE BENEFITS, AND CITIZENSHIP STATUS

**DOI:** 10.1093/geroni/igae098.1732

**Published:** 2024-12-31

**Authors:** Bruna Lopez

**Affiliations:** Boston College, Chestnut Hill, Massachusetts, United States

## Abstract

Hispanics play a significant role in the self-employment landscape among older workers, yet research on this subject often ignores intragroup heterogeneity. Investigating these intragroup differences may highlight differential access to safety-net programs (e.g., health insurance, retirement savings programs) and economic opportunities (e.g., business incorporation). In response, this study utilized data from the Current Population Survey Annual Social and Economic Supplement, obtained from IPUMS-CPS, to assess differences in incorporated (I-SE) and unincorporated (U-SE) self-employment rates among those 50 to 64 years old by seven Hispanic subgroups within the U.S. (Mexican, Puerto Rican, Cuban, Dominican, Salvadoran, Central and South American, and Other Hispanic). Using pooled data between 2017 and 2022 to reduce sampling noise, we estimated health insurance access, retirement savings program access, and citizenship status by subgroup and incorporation status. We found notable differences by subgroup in overall self-employment rates (e.g., Cubans had the highest rates: 5% I-SE, 6% U-SE%; Puerto Ricans had the lowest rates: 2% I-SE, 2% U-SE). Salvadorans had the lowest health insurance coverage rates (48% I-SE, 58% U-SE). Those falling within the “Other Hispanic” category, including Spaniards, participated the most in workplace retirement savings programs (26% I-SE, 7% U-SE). Excluding Puerto Ricans, who are U.S. citizens, Dominicans in unincorporated self-employment (46% citizens) and Salvadorans in incorporated self-employment (41% citizens) were the least likely to hold citizenship status. These findings identify important gaps in access to health insurance and retirement savings programs, as well as more lucrative incorporated self-employment opportunities, by subgroup.

